# Humoral and Cellular Immunity Are Significantly Affected in Renal Transplant Recipients, following Vaccination with BNT162b2

**DOI:** 10.3390/vaccines11111670

**Published:** 2023-10-31

**Authors:** Asimina Fylaktou, Stamatia Stai, Efstratios Kasimatis, Aliki Xochelli, Vasiliki Nikolaidou, Anastasia Papadopoulou, Grigorios Myserlis, Georgios Lioulios, Despoina Asouchidou, Maria Giannaki, Evangelia Yannaki, Georgios Tsoulfas, Aikaterini Papagianni, Maria Stangou

**Affiliations:** 1 Department of Immunology, National Histocompatibility Center, Hippokration General Hospital, 54642 Thessaloniki, Greece; fylaktoumina@gmail.com (A.F.); aliki.xochelli@gmail.com (A.X.); basoniko@hotmail.com (V.N.); deasouh@gmail.com (D.A.); 2 Department of Nephrology, Hippokration Hospital, 54642 Thessaloniki, Greece; staimatina@yahoo.gr (S.S.); frasci@outlook.com.gr (E.K.); pter43@yahoo.gr (G.L.); aikpapag@auth.gr (A.P.); 3 School of Medicine, Aristotle University of Thessaloniki, 54124 Thessaloniki, Greece; tsoulfas@auth.gr; 4 Hematology Department-Hematopoietic Cell Transplantation Unit, Gene and Cell Therapy Center, “George Papanikolaou” Hospital, 57010 Thessaloniki, Greece; papada_1@hotmail.com (A.P.); giannakimar@hotmail.com (M.G.); eyannaki@uw.edu (E.Y.); 5 Department of Transplant Surgery, Hippokration Hospital, 54642 Thessaloniki, Greece; gmyserlis@gmail.com

**Keywords:** renal transplantation, SARS-CoV-2, vaccination, humoral response, cellular response, ELISpot

## Abstract

Background. Renal transplant recipients (RTRs) tend to mount weaker immune responses to vaccinations, including vaccines against the novel severe acute respiratory syndrome coronavirus 2 (SARS-CoV-2). Methods. Humoral immunity was assessed using anti-receptor binding domain (RBD) and neutralizing antibodies (NAb) serum levels measured by ELISA, and cellular immunity was assessed using T-, B-, NK, natural killer-like T (NKT)-cell subpopulations, and monocytes measured by flow cytometry, and also specific T-cell immunity, at predefined time points after BNT162b2 vaccination, in 57 adult RTRs. Results. Administration of three booster doses was necessary to achieve anti-RBD and NAb protective levels in almost all patients (92.98%). Ab production, at several time points, was positively correlated with the corresponding renal function and inversely correlated with hemodialysis vintage (HDV) and treatment with mycophenolic acid (MPA). A gradual rise in several cell subpopulations, including total lymphocytes (*p* = 0.026), memory B cells (*p* = 0.028), activated CD4 (*p* = 0.005), and CD8 cells (*p* = 0.001), was observed even after the third vaccination dose, while a significant reduction in CD3+PD1+ (*p* = 0.002), NKT (*p* = 0.011), and activated NKT cells (*p* = 0.034) was noted during the same time interval. Moreover, SARS-CoV-2-specific T-cells were present in 41% of the patients who were unable to develop Nabs, and their positivity rates four months after the second dose were in inverse correlation with monocytes (*p* = 0.045) and NKT cells (*p* = 0.01). Conclusions. SARS-CoV-2-specific T-cell responses preceded the humoral ones, while two booster doses were needed for this group of immunocompromised patients to mount a protective immune response.

## 1. Introduction

Kidney transplantation is the treatment of choice for end-stage kidney disease, as it restores renal function and subsequently the associated metabolic disorders and complications. Patients’ immune profile after kidney transplantation is expected to be reinstated, albeit possibly, the procedure cannot fully reverse a series of chronic kidney disease (CKD)-induced immunological changes, which are primarily characterized by premature T-cell system aging. Indeed, lymphocyte functional abnormalities and subpopulation variations, mostly with regard to the presence of a suboptimal thymic output combined with the homeostatic proliferation of naïve T-cells, have been observed in these patients. This condition has mainly been attributed to the accumulation of irreversible uremia-associated epigenetic alterations [1]. As anticipated, the combination of an impaired immune system status with chronic immunosuppressive treatment (IST) leads to increased morbidity and mortality due to infectious complications [2,3], as well as blunted responses to vaccinations, in terms of seroconversion rates, antibody (Ab) titers and protective immunity duration [4]. 

The case is not much different when it comes to the SARS-CoV-2 virus, with renal transplant recipients (RTRs) presenting a higher infection incidence and an increased possibility of adverse disease outcomes (especially early post-transplantation) compared to the general population [5,6,7,8]. Humoral responses to anti-SARS-CoV-2 vaccination are also compromised, as these patients tend to have lower Ab positivity rates and concentrations than healthy individuals [9], combined with a poorer and less effective specific T-cell immunity activation [10,11]. IST, and particularly mycophenolate mofetil (MMF) administration, older age, lower estimated glomerular filtration rate (eGFR), and presence of comorbidities are only a few of the factors implicated in impaired immunogenicity status in this patient population [9,10,11,12]. 

Until now, a considerable number of different vaccine platforms against SARS-CoV-2 have been developed [13,14]. BNT162b2 is a nucleoside-modified messenger RNA (mRNA) vaccine, developed by Pfizer-BioNTech. The incorporated mRNA molecule is responsible for an encoding spike (S) protein. Lipid nanoparticles are used as carriers, in order to protect the molecules and facilitate their entrance into target cells. Introduction of the produced peptide on the cell surface of antigen-presenting cells can induce robust CD4+ and CD8+-mediated immune responses [15,16,17,18], while SARS-CoV-2-specific responses have been reported in both healthy individuals and hematopoietic stem cell transplant recipients [19,20,21]. According to the National Vaccination Committee, the first and the second vaccine doses are administered in a 21-day interval, the third one can be given at least 3 months after dose 2, and the fourth (second booster dose) at least 4 months following the third [22,23,24]. 

The present study aimed to prospectively assess the humoral and cellular immune response and the phenotypic alterations of lymphocytes and monocytes after anti-SARS-CoV-2 vaccination with BNT162b2 in RTRs.

## 2. Materials and Methods

### 2.1. Patients 

Recruited patients were Greek Caucasian RTRs. The study received institutional review board approval. All patients were informed about the study by their responsible physician and signed informed consent before entering the study.

Patients included in the study were adults (≥18 years old), RTRs who had been transplanted at least 3 months before enrolment, were being followed in the outpatients’ clinic, and were under stable immunosuppressive treatment (IST) during the whole study period. All participants had a negative history of severe acute respiratory syndrome coronavirus 2 (SARS-CoV-2) infection and undetectable anti-SARS-CoV-2 antibodies. 

Patients with active systemic disease, microbial or viral infection (during the last trimester), acute cellular or humoral rejection events (during the semester preceding their recruitment), or solid-organ or hematopoietic cell malignancies (during the past two years) or recent (during the past two years) administration of chemotherapeutic regimens or rituximab were excluded from the study. 

A total number of 57 individuals participated in the study.

### 2.2. Schedule of the Study

Our research was a prospective observational study, with the patient pool comprising RTRs being followed in the outpatient clinics of the AUTH Nephrology Department and Department of Transplant Surgery. 

**Figure 1:**
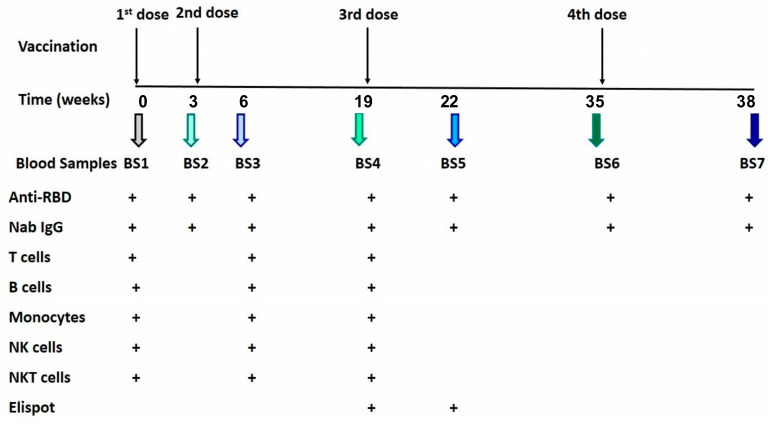
Schedule of the study. The upper panel demonstrates the vaccination program. In the lower part, the figure presents the timetable of blood samplings and the parameters measured.

Vaccinations were carried out based on the time schedule of the National Vaccination Committee (first dose: t_0_; second dose: 21 days after the first; third dose: 4 months after the second; fourth dose: 4 months after the third dose). The time points at which blood samplings (BS) were performed are presented in Supplementary Table S1 and the schedule of the study is elucidated in Figure 1.

The collected samples were analyzed for:

I. Anti-RBD and NAb IgG concentrations with the use of chemiluminescence immunoassay (CLIA). Analysis was performed in all blood samples (BS1–BS7).
II. CD4, CD8, and B lymphocytes and their subpopulations, monocytes, NK, and NKT cells, using flow cytometry, were counted at BS1, BS3, and BS4.
III. SARS-CoV-2-specific T-cell responses with enzyme-linked immunosorbent spot (ELISpot) were estimated at BS4 and BS5, only for those patients who had failed to develop protective NAb titers by BS3.

During the first patient visit, additional data were recorded, including demographic, clinical, and laboratory information. 

### 2.3. Laboratory Methods

#### 2.3.1. Flow Cytometry

Heparinized venous blood samples from RTRs were collected at the pre-defined time points and processed for the evaluation of proportions and counts of total lymphocytes, B and T lymphocytes, natural killer cells (NK), natural killer-like T-cells (NKT), and monocytes. The expression of CD27, CD38, IgD, and IgM was assessed on B lymphocytes, CD38 and HLA-DR were assessed on both CD4 and CD8 T lymphocytes, and the expression of CD14 and CD38 on monocytes. Finally, CD3, CD16, and CD56 were also estimated on T lymphocytes, to recognize NK, NKT cells, and PD1+ on CD3+ T-cells. All receptors were detected by using a cytometer (Navios Flow Cytometer, Beckman Coulter, Brea, CA, USA). The monoclonal antibodies used and their combinations are described in Supplementary Table S2.

Based on the search of the above receptors, the described lymphocyte subpopulations were:

For B lymphocytes:

I. Β-lymphocytes (CD45+CD19+);
II. Naïve Β-lymphocytes (CD45+CD19+CD27-);
III. Marginal B-lymphocytes (CD45+CD19+IgD+CD27-);
IV. Transitional B-lymphocytes (CD45+CD19+CD24+CD38^high^);
V. Memory Β-lymphocytes (CD45+CD19+CD27+);
VI. Plasmablasts (CD45+CD19+CD27+CD38^high^, divided further into non-switched plasmablasts (IgD+) and switched plasmablasts (IgD-).

For Τ lymphocytes:

I. Helper T lymphocytes (CD4+);
II. Activated CD4 (CD4+CD38+/HLA-DR+);
III. Cytotoxic T lymphocytes (CD8+);
IV. Activated CD8 (CD8+CD38+/HLA-DR+);
V. CD3+PD1+natural killer cells (CD45+CD3-CD56+);
VI. Natural killer-like T-cells (NKT cells) (CD45+CD3+CD56+) and activated NKT (CD45+CD3+CD56+CD38+ HLA-DR+);
VII. Monocytes (CD45+CD14+) and activated monocytes (CD45+CD14+CD38+ HLA-DR+).

Gating strategies for B- and T-lymphocyte subpopulations and monocytes are described in Supplementary Figures S1–S4.

#### 2.3.2. Assessment of Anti-RBD and NAb Levels

The SARS-CoV-2 S-RBD IgG (CLIA) assay Maglumi™ 2000 Plus (New Industries Biomedical EngineeringCo., Ltd. (Snibe), Shenzhen, China), used for the measurement of anti-RBD IgG Ab levels, is an indirect chemiluminescence immunoassay. Magnetic microbeads coated with S-RBD recombinant antigen are mixed with the sample and incubated, forming immune complexes. N-(4-Amino-Butyl)-N-Ethyl-Isoluminol (ABEI) labeled with anti-human IgG antibody is added and incubated to form complexes. The light signal produced by the chemiluminescent reaction is measured as relative light units (RLUs), which is proportional to the concentration of S-RBD IgG present in the sample.

The CLIA assay that we utilized for the detection of Nabs is a competitive chemiluminescence immunoassay. Magnetic microbeads coated with ACE2 antigen and ABEI labeled with recombinant S-RBD antigen are mixed with the sample and incubated. SARS-CoV-2 neutralizing antibody present in the sample competes with the ACE2 antigen immobilized on the magnetic microbeads for the binding recombinant S-RBD antigen labeled with ABEI. The light signal is measured as relative light units (RLUs), which is inversely proportional to the concentration of the SARS-CoV-2 neutralizing antibody. 

Patients who reached the cut-offs of 1.0 AU/mL for anti-RBD Abs and 0.3 AU/mL for NAbs were considered responders to vaccination, in terms of humoral immunity (according to the manufacturer).

#### 2.3.3. Assessment of SARS-CoV-2-Specific T-Cell Levels

SARS-CoV-2-specific T-cells were measured as previously described [21]. Peripheral blood mononuclear cells (PBMCs) were stimulated with overlapping 15-mer PepMixes of the full-length spike protein (JPT Peptide Technologies) and the secretion of interferon-gamma (IFN-γ) was measured by ELISpot. Spot-forming cells (SFCs) were counted using an Eli.Scan ELISpot scanner (A.EL.VIS) using Eli.Analyse software V6.2.SFC. SARS-CoV-2 spike-specific T-cells were expressed as SFCs per input cells and the response was considered positive if the total cytokine-producing SFCs against spikes were ≥30 per 5 × 10^5^ PBMCs.

### 2.4. Statistical Analysis

Statistical analysis was performed with the use of IBM SPSS 26.0 (SPSS Inc., Chicago, IL, USA). Results with a *p* value < 0.05 were considered statistically significant. Quantitative variables were examined for the presence of normal distribution using the Shapiro–Wilk test. The central tendency measures were the mean value +/− standard deviation (SD) for normally-distributed, and the median value (interquartile range, IQR) for non-normally distributed variables. OR was preferred for risk assessment and a confidence interval (CI) of 95% was set. The Friedman test was utilized for the comparison among more than two median values of successive results of the parameters under study (since the relevant variables did not follow a normal distribution). Afterwards, pairwise comparisons for all possible combinations were performed using the Wilcoxon test and the *p* values were subsequently adjusted with the use of the Bonferroni correction. Mean values (or medians in cases of non-normal distribution) of two independent groups were compared with the independent samples *t*-test (or with the Mann–Whitney U test, respectively). The presence of statistical dependence between the rankings of two normally distributed values was checked using the Pearson test, while for non-normally distributed values, the Spearman test was used. The possible correlation between two binary variables was assessed using the Chi-Square test and its degree was evaluated depending on the odds ratio (OR) value. 

## 3. Results

Patients’ demographics at time of vaccination are described in Supplementary Table S3.

### 3.1. Humoral Immunity Response to Vaccination

#### 3.1.1. Development of Anti-SARS-CoV2 IgG Antibodies

The kinetics of humoral response to vaccination at different time points is described in Table 1 and depicted in Supplementary Figure S5.

The difference recorded between Ab concentrations, as measured in successive blood samplings, was statistically significant in almost all cases. Anti-RBD Abs presented a gradual ascent in their titers until BS5, then a transient drop at BS6, and eventually a vertical rise at BS7. NAbs, on the other hand, seemed to drop until the administration of the third dose, which led to a statistically significant increase in their titers. This was also the case with dose 4, as it resulted in very notable changes in NAb concentration. Table 1 describes anti-SARS-CoV-2 IgG anti-RBD Ab and NAb serum levels and also signifies the rates of antibody concentration multiplication. 

Only 10.5% of vaccinated patients met the definition of responders at BS2; however, this proportion increased gradually at BS3, BS5, and BS7, to 47,4%, 85.96%, and 92.98%, respectively. On the contrary, neutralizing humoral responses were considerably delayed, as 31.58%, 10.53%, 70.18%, and 92.98% of patients developed protective NAbs, at BS3, BS4, BS5, and BS7, respectively (Supplementary Table S4).

#### 3.1.2. Parameters Associated with Antibody Response to Vaccination

Regarding patient parameters associated with anti-RBD Ab and NAb concentrations, we found that eGFR, hemodialysis vintage (HDV), and treatment with mycophenolic acid (MPA) were the most important ones. Patients who, after renal transplantation, were on a lower dosage of MPA, and retained better renal function, with higher eGFR levels, were also more able to respond to vaccination, even only after the second vaccination dose. Also, patients who stayed on hemodialysis for a shorter time had more possibilities to respond to vaccination (Supplementary Tables S5 and S6). 

### 3.2. Cellular Immunity Response to Vaccination

#### 3.2.1. Cell Subpopulation Changes during Follow up

**Figure 2:**
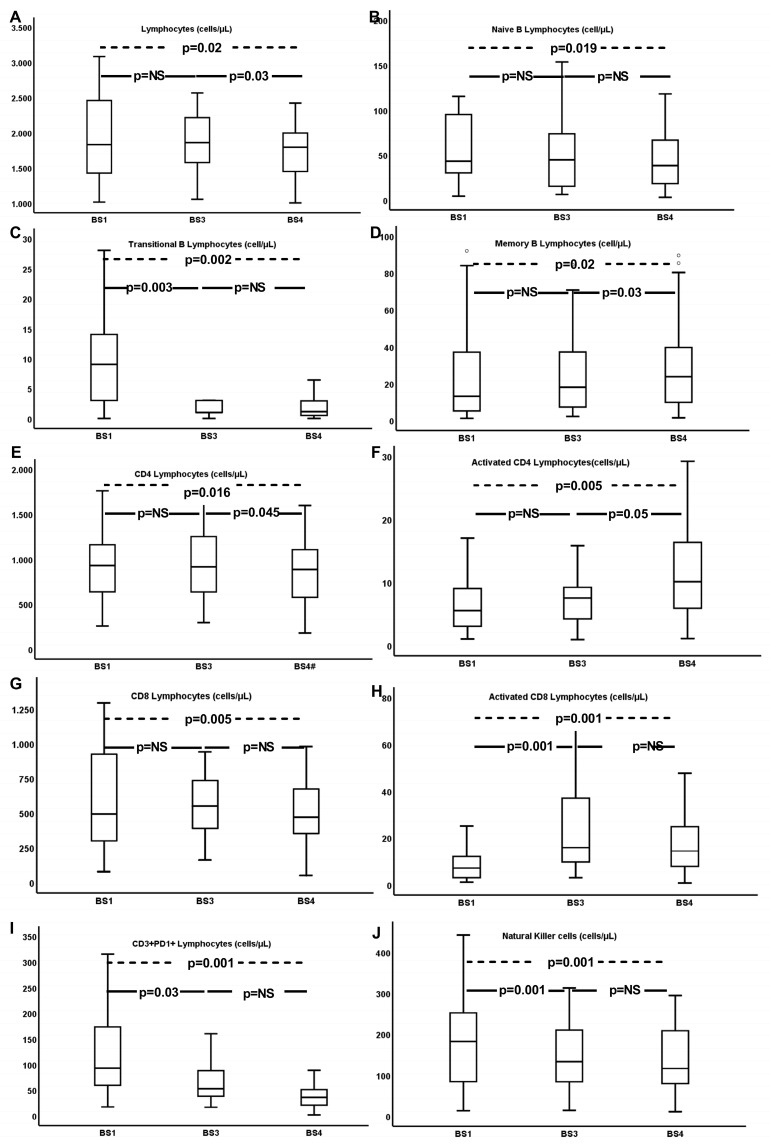
The most important changes in lymphocyte subtypes, during the time period, from BS1 towards BS3 and BS4 in the whole cohort of patients. Changes in total lymphocytes (**A**), naïve (**B**), transitional (**C**), memory (**D**) B lymphocytes, CD4 (**E**) and activated CD4 (**F**) lymphocytes, CD8 (**G**) and activated CD8 (**H**) lymphocytes, CD3+PD1+ (**I**) and natural killer cells (**J**).

Recorded changes in total lymphocytes and their subpopulations are described in Table 2 and depicted in Figure 2. There was a notable increase of total lymphocytes, memory B-cells, activated CD4, CD8, monocytes, and NK cells. CD3+PD1+ and NKT cells, on the other hand, were reduced significantly.

#### 3.2.2. Correlation between T- and B-Cell Immunity with Humoral Immunity 

As described above, 27 and 18 of the 57 patients developed protective anti-RBD and Nab titers by BS3, respectively. In these patients, lymphocyte changes were also recorded at the same time point, affecting total lymphocytes and subpopulations. More specifically, B-cells were increased, with a clear shift to marginal and memory cells, against a significant reduction of plasmablasts and transitional B-cells. Regarding T lymphocytes, both CD4 and CD8 cells were increased, with changes affecting mainly activated T lymphocytes. Interestingly, PD1+, NKT, and activated NKT cells were reduced (Table 3). 

**Figure 3:**
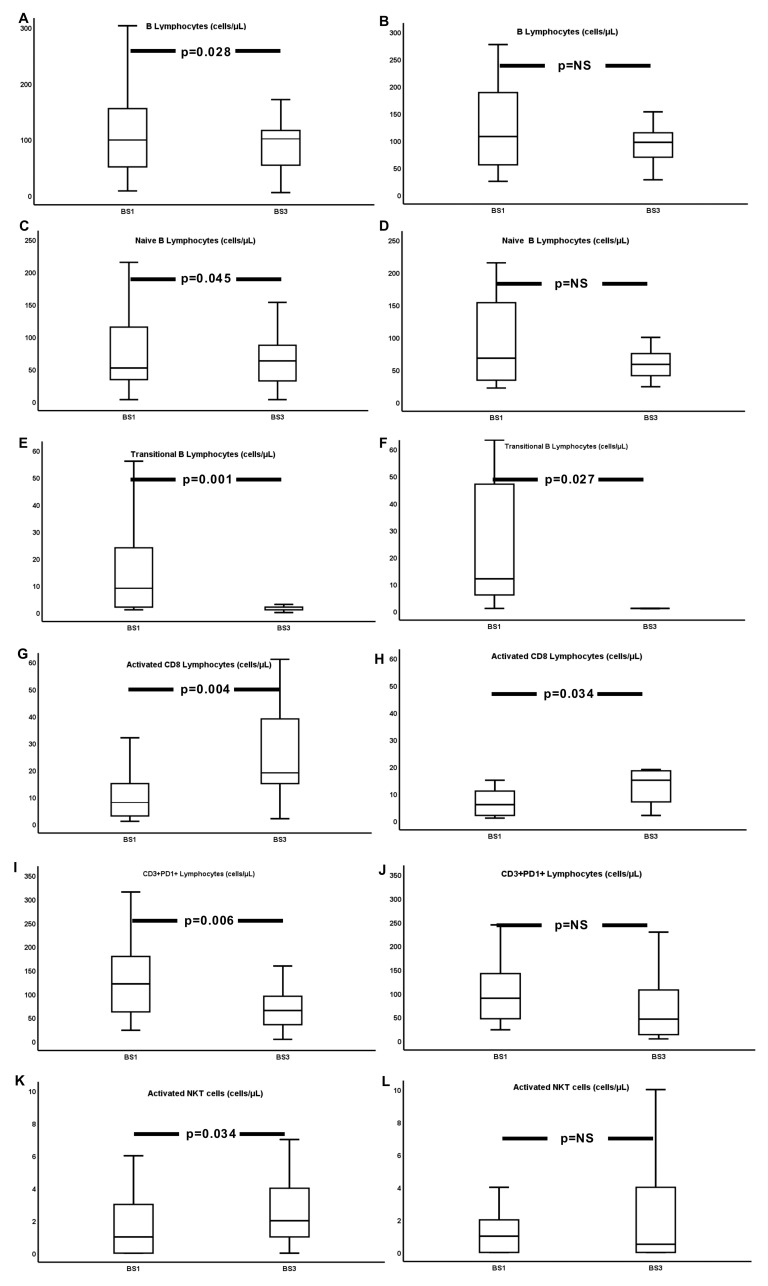
Changes from BS1 to BS3 in total (**A**,**B**), naïve (**C**,**D**) and transitional (**E**,**F**) B lymphocytes, activated CD8 (**G**,**H**), CD3+PD1 (**I**,**J**) and activated NKT (**K**,**L**) cells in BS3—anti-RBD (+)—and in BS3—anti-Nab (+)—patients, respectively.

Figure 3 demonstrates the most important alterations in B and T lymphocytes in anti-RBD (+) and anti-Nab (+) patients at BS3.

In NAb-negative individuals, similar, yet less-significant alterations were observed in B lymphocytes, which demonstrated a significant reduction in plasmablasts. However, regarding the T-cell compartment, different subpopulations were increased, including activated CD8, monocytes and NKT cells, but not activated CD4 cells (Supplementary Table S7). 

#### 3.2.3. SARS-CoV-2-Specific T-Cell Response

**Figure 4:**
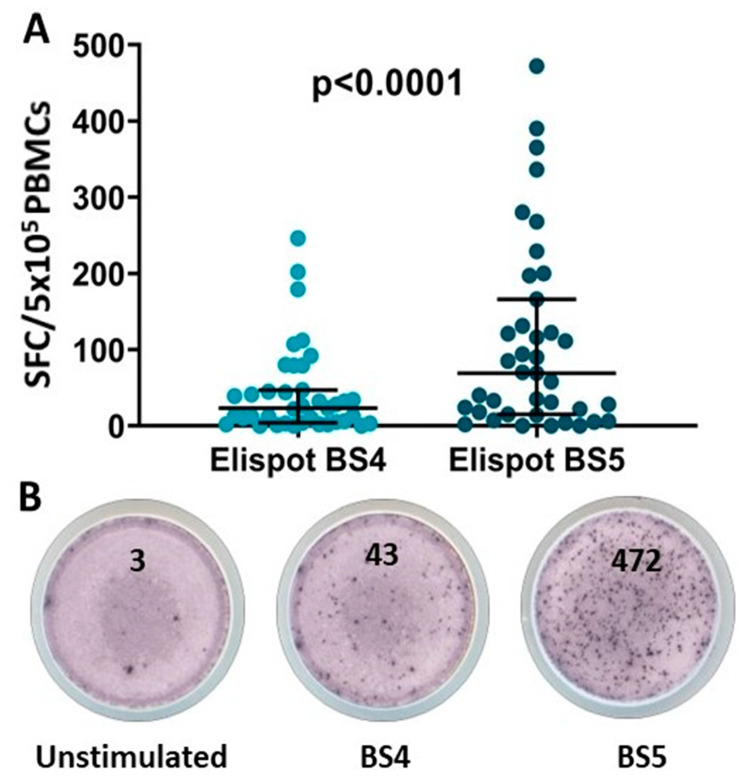
(**A**) SARS-CoV-2-specific T-cell levels at time points BS4 and BS5, in NAb (-) patients. (**B**) A representative ELISpot of a patient’s SARS-CoV-2-specific T-cells, before and after the third dose. Unstimulated cells served as a negative control.

In order to have a better understanding of the major components of the adaptive immunity of RTRs post-COVID-19 vaccination, we assessed also the spike-specific T-cell immunity before and after the third dose (BS4 and BS5, respectively) in subjects with almost undetectable NAbs after receiving the first two vaccination doses. Interestingly, 16/39 of patients (41%) with undetectable NAb levels after the second dose (BS4) demonstrated protective T-cell immunity (≥30 SFCs/500.000 PBMCs) with 63(68) SFC/5 × 10^5^ PBMCs circulating SARS-CoV-2-specific T-cells, whereas following the third vaccination dose (BS5), 25/39 of non-responders (64.1%) developed specific T-cell responses (chi-square 4.16, *p* = 0.04) of higher magnitude, 21(179) SFC/5 × 10^5^ PBMCs (*p* < 0.0001) (Figure 4). 

**Figure 5:**
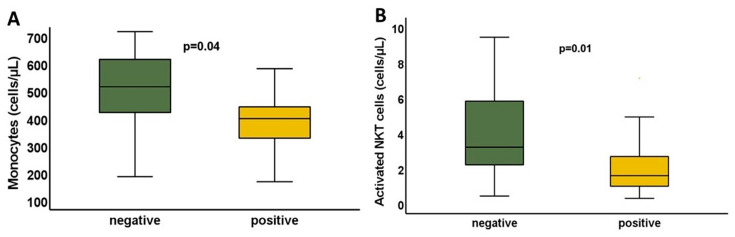
The concentration of Monocytes (**A**) and activated NKT cells (**B**) in the patients tested for SARS-CoV-2-specific T-cell response at BS4. The grafts demonstrate significant differences in monocytes and activated NKT cells between responders and non-responders.

Specific T-cell responses were increased in patients with lower monocyte numbers (405.99 ± 133.54 vs. 516.53 ± 179.882 cells/μL, *p* = 0.045, t = 2.074) and lower activated NKT cells (1.6(2) vs. 3.21(4) cells/μL, *p* = 0.01 for patients with or without T-cell response, respectively) in BS4 (Figure 5). 

No other parameters (including cell subpopulations, age, eGFR levels, transplantation, and dialysis vintage, retransplantation, pre-emptive dialysis or diabetes mellitus status, tacrolimus, and cyclosporine A levels), neither in BS4 nor in BS5, seemed to correlate with specific T-cell responses.

## 4. Discussion

In the present study, we evaluated the humoral and cellular response to SARS-CoV-2 in RTRs, by estimating serum levels of anti-RBD Abs and NAbs, certain subtypes of lymphocytes and monocytes, and also, SARS-CoV-2-specific T-cell responses. All measurements were performed at definite time points following doses of vaccination. All RTRs had four doses of vaccination, and the scheduled estimation of humoral and cellular immunity covered the response rate from the first to the third weeks after the fourth vaccination dose. 

Our results show a significant increase in anti-RBD levels even after the second vaccination dose, although the development of adequate NAbs was significantly delayed. Our results are in accordance with previous studies, which showed that vaccine-induced immune response against SARS-CoV-2 in RTRs was significantly compromised compared to that of healthy controls, regardless of the vaccine type they received [25]. Many authors have reported remarkably lower Ab levels in that group of individuals, and indeed, almost one third of RTR patients develop adequate serum antibody levels and could be characterized as responders after the administration of two mRNA vaccine doses [26,27,28,29]. In our study, we noticed an induction of humoral response after the third BNT162b2 dose. In particular, both serum anti-RBD Ab and NAb levels increased after the third vaccination dose, reaching more than 70 times and 8 times the threshold levels for anti-RBDs and for NAbs, respectively, among the responders. These levels slightly waned over time, justifying the fourth dose, which was proved necessary, in order to achieve maximum levels of the humoral response. McEvoy et al. observed a similar reduction in the median anti-RBD Ab levels three months after dose 3, although the percentage of RTRs with sufficient Ab development remained stable [30]. Importantly, in our study, the administration of the fourth BNT162b2 dose substantially improved the humoral immune response, with nearly all participants producing remarkably higher anti-SARS-CoV-2 Abs a month later.

Our analysis also demonstrates that Ab development was positively associated with the individual’s eGFR levels, while both hemodialysis vintage prior to RTx and current treatment with MPA had a significant negative impact on the patients’ response to vaccination. These findings were consistent with the current literature [27,31,32]. A 0.92% reduction in seropositivity for every 1% increase in MPA administration has been reported, and attributed to the MPA potential inhibitory effect on B- and Th-cell proliferation [9]. In the same context, the use of everolimus, instead of MPA, in combination with lower calcineurin inhibitor dosages, was linked to a more robust Ab production [33]. According to other researchers, a large number of additional parameters are related to a poorer humoral immunity activation, including increasing age [31,32,34], shorter duration between transplantation and vaccination (Ab acquisition is especially difficult during the first post-transplantation year), lower body mass index (BMI) [28], presence of anemia [27], female gender [26], diabetic nephropathy [32], deceased donor state, recent exposure to rituximab or antithymocyte globulin [34], and insufficient cellular response rate and IFN-γ production [35]. 

Concerning alterations of cellular immunity, total lymphocyte count was significantly increased following the second dose of vaccination, both at BS3 and at BS4 (3 weeks and 4 months after dose 2, respectively). B-cells and some of their subpopulations (namely memory and naïve B-cells), were also significantly elevated at the same time points. On the contrary, plasmablasts and transitional B-cells were reduced, presenting an early decline after the second vaccine dose.

As for T-cells, CD4 and CD8 cells followed a similar process, indicating a transient rise, immediately after the second dose, which subsequently dropped 3–4 months later. Very interestingly, though, despite this reduction in the CD4 and CD8 populations, the respectively activated subpopulations showed an early increase, which remained stable. In a similar way, NK cells, activated NKT cells, and monocytes were also increased and remained at higher levels, and only the CD3+PD1+ subpopulation was gradually and significantly reduced after two vaccination doses. 

The failure to achieve a protective immune response through vaccination in a great number of RTRs has been attributed to their insufficient ability to produce adequate concentrations of vaccine-specific CD4+ T-cells and B-cells, due to IST. Interestingly, in those patients, most antigen-specific B-cells are identified in the pre-switch and the naïve compartment [36]. According to the findings of Sattler et al., even though the percentage of RTRs mounting spike-specific CD4+ responses is comparable to that of healthy individuals and dialysis patients, they have significantly lower proportions of spike-specific Th-cells, combined with impaired effector cytokine production and memory differentiation, while in the great majority of RTRs, spike-specific CD8+ T-cells are not even detectable [11]. Other researchers have recently studied the alterations in the absolute numbers of B-cell and several T-cell subsets after SARS-CoV-2 vaccination in RTRs, and found a reduction in CD8+ and an increase in CD4+ and NKT-cell concentrations, together with an ascent of IL-2+, IL-4+, and polyfunctional T-cells, while the memory/effector subset composition remained unaltered [37,38]. 

The antigen-specific memory B-cell response is of great importance to the adequacy of mRNA vaccines and has been correlated with previous memory B-cell populations [39]. Our patients showed an increase in memory B-cells, together with active CD4, CD8 cells, and monocytes. 

B lymphocytes are the key mediators of antibody production, closely synergizing with T-cells in order to perform that function. Therefore, it was of no surprise that we observed differences in lymphocyte count fluctuations between responders and non-responders to vaccination (as defined based on anti-RBD and NAb positivity). Anti-RBD Ab-positive patients presented some differences from our total sample regarding cell subpopulation characteristics in given time points. They displayed a significant rise of B-cells, which was mainly attributed to the expansion of the naïve cell compartment, as the other B-cell subpopulations decreased. Memory B-cells were stable, as were activated CD4 cells and monocytes, while activated CD8 and NK cells were significantly increased. Individuals with protective NAb titers, on the other hand, displayed an increase in total lymphocyte, marginal B-cell, and CD3+ T-cell counts (not recorded in the whole cohort), while their numbers of CD3+CD4+ T-cells, activated NKT, and activated monocytes practically remained unaltered. Anti-RBD Ab development has been previously linked to significantly higher numbers of B-cells and spike-reactive CD4+ cells, as well as to greater CD8+ percentages. Pre-vaccination concentrations of antigen-non-specific memory and transitional B-cells were found to have an important role in spike-specific memory B-cell and plasmablast numbers following vaccination [36,40]. 

A key finding of our study is that robust SARS-CoV-2-specific T-cell responses were elicited in a considerable percentage of RTRs who had failed to develop NAbs after two-dose vaccination, indicating that the development of SARS-CoV-2-specific T-cell immunity preceded the relevant humoral immunity in RTR vaccinated individuals. The observed earlier development of T-cell immunity is in line with previous reports in hematopoietic stem cell transplant recipients [19], which could be interpreted by the supporting role of CD4+ cells in B-cell maturation and therefore neutralizing antibody production following SARS-CoV-2 vaccination [40]. In a different context, we previously showed that the early development of SARS-CoV-2-specific T-cell immunity positively correlates with a favorable outcome in patients with COVID-19 [21] and that in patients with severe COVID-19 who received SARS-CoV-2-specific T cells, the higher in vivo expansion of SARS-CoV-2-STs over patients receiving standard care only had a major impact on a favorable outcome [41].

Underscoring the importance of the booster doses in RTRs, protective cellular immune response was mounted following the third vaccination dose in 64% of the non-responders after the first two doses. The assessment of SARS-CoV-2-specific T-cell immunity only in NAb-negative individuals at two time points is a potential limitation of our study. 

We focused, however, on this particular patient cohort, consisting of immunocompromised individuals with delayed development of humoral responses, in whom booster vaccine doses may provide the highest benefit. Given their severely immunocompromised status, resulting from a combination of IST administration and their failure to restore CKD-associated immunological impairments, it would be reasonable to presume that RTRs could possibly benefit from the implementation of a different, more intensive anti-SARS-CoV-2 immunization program. However, precise strategies that would increase vaccination effectiveness still remain to be proposed. The innovation of the present research lies in the inclusion of a very wide spectrum of cellular subsets, as well as in the attempt to closely monitor their fluctuations at pre-defined post-vaccination time points. The latter, alongside with the knowledge regarding the exact function of each subpopulation and their interactions, would be useful in the design of new vaccination schemes, adapted to the special needs of this patient group. A rough assessment of our results proves the undoubtable value of booster doses, mostly underlining the importance of the second and the third BNT162b2 dose for effectively increasing (or at least maintaining) antibody concentrations and immune cell numbers to acceptable levels. Interestingly, it seems that the time interval of 4 months between dosages is possibly quite long and results in a considerable decline of humoral and cellular immunity components. In that context, shorter periods between vaccinations, or even greater dosages, could be considered in an attempt to improve anti-SARS-CoV-2 vaccination effectiveness among RTRs.

## 5. Conclusions

Our study clearly demonstrated a significant and relatively early response in cellular immunity following vaccination with BNT162b2 against SARS-CoV-2 in RTR, assessed by the rate of lymphocyte and monocyte activation. The humoral response was delayed, requiring the administration of two booster doses. Therefore, there is evidence that cellular immunity may reach protective stages, even in patients who failed to develop adequate levels of NAbs, as indicated by the SARS-CoV-2-specific T-cell responses. However, we need to further evaluate whether T-cell activation is sufficiently and appropriately protective to patients. 

## Figures and Tables

**Table 1:** (A) Serum concentrations of anti-SARS-CoV-2 IgG RBD Abs and NAbs in all time points; (B) Anti-SARS-CoV-2 Ab concentrations’ central tendency measures as compared to anti-RBD Ab and NAb positivity threshold values (times × the threshold, includes only patients with Ab concentrations above the cut-off value). Antibody levels at baseline, BS1, are not described, as they were all 0 AU/mL.

**A**	**BS2**	**BS3**	**BS4**	**BS5**	**BS6**	**BS7**	***p***
**Anti-RBD Abs (AU/mL)**	0.37 (0.25)	0.84 (8.04)	2.26 (8.21)	64.13 (134.53)	40.72 (73.31)	462.91 (857.38)	<0.001
**NAbs (AU/mL)**	0.14 (0.71)	0.11 (0.31)	0.07 (0.19)	1.711 (3.79)	1.17 (2.99)	8.56 (15.01)	NS
		**BS2–BS3**	**BS3–BS4**	**BS4–BS5**	**BS5–BS6**	**BS6–BS7**	
**Anti-RBD Abs**	*p*	<0.001	NS	<0.001	<0.001	0.001	
		<0.001 *	NS *	0.00381 *	0.00573 *	0.015 *	
**NAbs**		0.043	0.017	<0.001	0.001	0.002	
		NS *	NS *	<0.001 *	0.015 *	0.03 *	
**Β**	**BS2**	**BS3**	**BS4**	**BS5**	**BS6**	**BS7**	***p***
**Anti-RBD Abs**	4.37 (41.0)	9.22 (40.1)	3.37 (9.4)	72.43 (120.1)	60.32 (77.1)	465.50 (841.1)	NS
**NAbs**	2.69 (0.43)	1.88 (3.30)	1.97 (1.48)	8.87 (15.87)	6.01 (10.23)	31.13 (47.32)	NS
		**BS2–BS3**	**BS3–BS4**	**BS4–BS5**	**BS5–BS6**	**BS6–BS7**	
**Anti-RBD Abs**		0.046	0.039	<0.001	0.001	0.002	
		NS *	NS *	<0.001 *	0.015 *	0.03 *	
**NAbs**		NS	NS	NS	0.001	0.003	
		NS *	NS *	NS *	0.01 *	0.03 *	

*: the *p*-values were corrected using the Bonferroni test. Abbreviations: anti-RBD Abs, anti-receptor binding domain antibodies; BS, blood sampling; Nabs, neutralizing antibodies; and NS, non-significant. The central tendency measures used are the median value (MED) (interquartile range, IQR).

**Table 2:** Concentrations of cell subpopulations (cells/μL) at different time points in the whole cohort of patients.

	BS1	BS3	BS4	*p* *	*p* (BS3 vs. BS1) ^†^	*p* (BS4 vs. BS3) ^†^
**WBC**	7650 (2600)	7600 (1850)	8000 (1750)	NS	NS	NS
**Lymphocytes**	1597 (1169)	1881 (864)	1778 (1004)	0.026	NS	0.03
**B-cells**	70.5 (106)	75 (88)	81 (89)	NS	NS	NS
**Naïve B-cells**	48.5 (77)	50 (56)	48.5 (78)	0.019	NS	NS
**Transitional B-cells**	3 (11)	1 (2)	4 (6)	0.002	0.003	NS
**Marginal B-cells**	10.7 (11.4)	10.6 (9.7)	13.8 (11.8)	NS	NS	NS
**Memory B-cell**	15.5 (33)	19 (25)	24 (28)	0.028	NS	0.03
**Plasmablasts**	0.95 (4)	0 (0.4)	0.1 (0.8)	NS	<0.001	NS
**CD3+ T-cells**	1305 (1022)	1546 (963)	1444 (854)	0.004	NS	NS
**CD3+CD4+ T-cells**	832 (584)	948 (646)	931 (522)	0.016	NS	0.045
**Activated CD4+ T-cells**	5 (5)	8 (7)	12 (12)	0.005	NS	0.05
**CD3+CD8+ T-cells**	494.5 (553)	560 (309)	486.5 (359)	0.005	NS	NS
**Activated CD8+ T-cells**	6 (10)	17.5 (26)	15.5 (17)	0.001	<0.001	NS
**CD3+PD1+ T-cells**	79 (94)	52 (51)	33.5 (30)	<0.001	0.03	NS
**CD3-CD56+ (NK) cells**	20.9 (59.55)	170.3 (137.4)	124.55 (132.73)	<0.001	<0.001	NS
**CD3+CD56+ (NKT) cells**	154.5 (163)	137 (155)	116 (132)	0.011	NS	NS
**Activated NKT cells**	1 (3)	2 (4)	0 (3)	0.034	0.021	NS
**Monocytes**	411 (360)	506 (228)	440.5 (209)	NS	NS	NS
**Activated monocytes**	211 (254)	358 (227)	324.5 (154)	NS	NS	NS

* *p* values calculated using the Friedman test; ^†^ *p* values calculated using the Wilcoxon test. Abbreviations: BS, blood sampling; CD, cluster of differentiation; NK, natural killer; NKT cells, natural killer-like T-cells; and NS, non-significant. The central tendency measures used are the median value (MED) (interquartile range, IQR).

**Table 3:** Lymphocytes and their subpopulation concentrations at BS1 and BS3 in the 27 and 18 patients who developed protective levels of anti-RBD Abs and NAbs at BS3.

	Anti-RBD Ab (+) Patients			NAb (+) Patients		
	(n = 27)			(n = 18)		
	BS1	BS3	*p*	BS1	BS3	*p*
**WBC**	7800 (2400)	7400 (2650)	NS	7200 (3525)	6700 (3500)	NS
**Lymphocytes**	1838 (1627)	2215 (947)	NS	1460.5 (1050)	2385.5 (1178)	0.005
**B-cells**	99 (118)	101 (74)	0.028	107.5 (156)	97 (50)	NS
**Naïve B-cells**	52 (93)	63 (64)	0.045	68 (127)	58.5 (38)	NS
**Transitional B-cells**	6 (20)	1 (1)	<0.001	9 (52)	1 (0)	0.027
**Marginal B-cells**	7.2 (11.2)	10.6 (9.9)	NS	6.9 (7.4)	13.55 (10.3)	0.032
**Memory B-cells**	26 (46)	24 (27)	NS	23 (48)	32 (32)	NS
**Plasmablasts**	2 (4.3)	0 (0.2)	<0.001	2 (5.9)	0 (0.1)	0.028
**CD3+ T-cells**	1545 (1224)	1856 (1064)	NS	1205.5 (893)	2152 (1114)	0.004
**CD3+CD4+ T-cells**	1049 (707)	1161 (711)	NS	810 (700)	1263.5 (1329)	0.009
**Activated CD4+ T-cells**	6 (6)	9 (8)	NS	3.5 (5)	10 (16)	0.024
**CD3+CD8+ T-cells**	663 (552)	644 (380)	NS	444.5 (512)	593.5 (345)	NS
**Activated CD8+ T-cells**	8 (13)	19 (29)	0.004	2.5 (10)	15(14)	0.034
**CD3+PD1+ T-cells**	121 (126)	65 (68)	0.006	89.5 (112)	45.5 (113)	NS
**CD3-CD56+ (NK) cells**	18.92 (33.54)	220.21 (177.4)	0.001	14.1 (147.89)	220.21 (285.23)	0.02
**CD3+CD56+ (NKT) cells**	149 (178)	105 (195)	0.032	126.5 (174)	46.5 (263)	NS
**Activated NKT cells**	1 (3)	2 (4)	0.034	1 (3)	0.5 (5)	NS
**Monocytes**	411 (360)	506 (228)	NS	NS	NS	NS
**Activated monocytes**	230 (2460)	348 (169)	NS	226 (433)	264 (194)	NS

Abbreviations: BS, blood sampling; CD, cluster of differentiation; NK, natural killer; NKT cells; natural killer-like T-cells; and NS, non-significant. The central tendency measures used are the median value (MED) (interquartile range, IQR).

## Data Availability

All data are available upon request.

## Supplementary Materials

The following supporting information can be downloaded at: https://www.mdpi.com/article/10.3390/vaccines11111670/s1, Table S1: Record of performed blood samplings; Table S2: Cluster of Differentiation (CD) detected on B- and T-lymphocytes; Table S3: Patient Demographics; Table S4: Presentation of the number (and percentage) of patients who developed serum levels of anti-RBD Abs and Nabs above the threshold and were regarded as responders, at the relevant time points; Table S5: Differences in eGFR levels at time of first vaccination dose, and in HDV, between responders and non-responders to BNT162b2; Table S6: Anti-SARS-CoV-2 antibody development with regards to MPA treatment; Table S7: Cell subpopulation concentrations (cells/μL) in BS1 and BS3 [NAb(-) patients in BS3; Figure S1: Gating strategy for CD3, CD4, CD8 and Activated CD4, Activated CD8 respectively; Figure S2: Gating strategy for CD3-CD56+ (Natural Killer – NK-) cells and Activated NK cells; Figure S3: Gating strategy for Monocytes and Activated Monocytes, CD3+CD56+ (NKT-cells) and Activated NKT-cells; Figure S4: Gating strategy for CD19+ cells, CD19+CD27- (naïve), CD19+CD27+ (memory), CD19+ CD27-IgD+ (marginal), CD19+CD27+CD38+(plasmablasts), CD19+CD27+IgD+ (non switched) and CD19+CD27+IgD-(switched) cells; Figure S5: Serum levels of anti-RBD Abs (A) and NAbs (B) at certain time points after vaccination, during follow up, BS2, BS3, BS4, BS5, BS6, BS7. Levels at baseline, BS1, are not demonstrated because they were zero.
