# Supplementary material for: Humoral and Cellular Immunity Are Significantly Affected in Renal Transplant Recipients, following Vaccination with BNT162b2
Source: Vaccines (Basel). 2023 Oct 31;11(11):1670. doi: 10.3390/vaccines11111670 (PMC10674678; doi:10.3390/vaccines11111670)
Supplement: Supplementary file 1 [file vaccines-11-01670-s001.zip › vaccines-2621520-supplementary.pdf]

Supplementary Material

Humoral and Cellular Immunity Are Significantly Affected in Renal Transplant Recipients, following Vaccination with BNT162b2

Asimina Fylaktou [1], Stamatia Stai [2,3], Efstratios Kasimatis [2], Aliki Xochelli [1], Vasiliki Nikolaidou [1], Anastasia Papadopoulou [4], Grigorios Myserlis [5], Georgios Lioulios [2,3], Despoina Asouchidou [1], Maria Giannaki [4], Evangelia Yannaki [4], Georgios Tsoulfas [3,5], Aikaterini Papagianni [2,3] and Maria Stangou [2,3,*]

[1] Department of Immunology, National Histocompatibility Center, Hippokration General Hospital, 54642 Thessaloniki, Greece; fylaktoumina@gmail.com (A.F.); aliki.xochelli@gmail.com (A.X.); basoniko@hotmail.com (V.N.); deasouh@gmail.com (D.A.)
[2] Department of Nephrology, Hippokration Hospital, 54642 Thessaloniki, Greece; staimatina@yahoo.gr (S.S.); frasci@outlook.com.gr (E.K.); pter43@yahoo.gr (G.L.); aikpapag@auth.gr (A.P.)
[3] School of Medicine, Aristotle University of Thessaloniki, 54124 Thessaloniki, Greece; tsoulfas@auth.gr
[4] Hematology Department-Hematopoietic Cell Transplantation Unit, Gene and Cell Therapy Center, "George Papanikolaou" Hospital, 57010 Thessaloniki, Greece; papada_1@hotmail.com (A.P.); giannakimar@hotmail.com (M.G.); eyannaki@uw.edu (E.Y.)
[5] Department of Transplant Surgery, Hippokration Hospital, 54642 Thessaloniki, Greece; gmyserlis@gmail.com
* Correspondence: mstangou@auth.gr; Tel.: +30-2310892030

Supplementary Table S1. Record of performed blood samplings

Blood Sample (BS) Number	Abbreviation	Time Points
1st	BS1	48 hours before dose 1
2nd	BS2	48 hours before dose 2
3rd	BS3	3 weeks after dose 2
4th	BS4	48 hours before dose 3 (Identifies 4 months after dose 2)
5th	BS5	3 weeks after dose 3

6th	BS6	48 hours before dose 4<br>(Identifies 4 months after dose 3)
7th	BS7	3 weeks after dose 4

Supplementary Table S2. Cluster of Differentiation (CD) detected on B- and T-lymphocytes

**CD markers**	**Fluorochrome**	**Clone**
CD45	KROME	J33
**B-cell markers**		
CD19	PC7	J4.119
IgD	FITC	IA6-2
CD27	ECD	IA4CD27
CD24	SN3	PerCP-Cy5.5
CD38	A750	LS198-4-3
**T-cell markers**		
CD3	FITC	UCHT1
CD4	APC	13B8.2
CD8	PC5.5	B9.11
CD56	A700	N901NKH-1
HLA-DR	PB	Immu-357
CD38	A750	LS198-4-3
PD1	PE	EH12.2H7

Supplementary Table S3. Patient Demographics

**N**	**57**
Male/Female	34/23
Age (years)	48(19)
Transplantation Vintage (years)	9.1(14)
Hemodialysis Vintage (months)	18(46)
eGFR (mL/min/1.73m^2)	54.3(20.3)
Tacrolimus levels (ng/mL)	6(1.5)
Cyclosporine levels (ng/mL)	268.5(222)

Preemptive Kidney transplantation	19.3% (11/57)
Retransplantation history	19.3% (11/57)
Diabetes Mellitus history	7% (4/57)
Treatment with mycophenolic acid	84.2% (48/57)

Supplementary Table 4. Presentation of the number (and percentage) of patients who developed serum levels of anti-RBD Abs and Nabs above the threshold and were regarded as responders, at the relevant time points

	**BS1**	**BS2**	**BS3**	**BS4**	**BS5**	**BS6**	**BS7**
**Anti-RBD Abs**	0%	6/57 (10.53%)	27/57(47.37%)	25/57(44.86%)	49/57(85.96%)	45/57(78.95%)	53/57(92.98%)
**NAbs**	0%	4/57 (7.02%)	18/57(31.58%)	6/57(10.53%)	40/57(70.18%)	39/57(68.42%)	53/57(92.98%)

Abbreviations: Anti-RBD Abs: anti-receptor binding domain antibodies; BS, blood sampling; and Nabs, neutralizing antibodies.

Supplementary Table S5. Differences in eGFR levels at time of first vaccination dose, and in HDV, between responders and non-responders to BNT162b2

**eGFR levels (ml/min/1.73m^2)**							
**Anti-RBD Ab titers**	**responders**	**non-responders**	**p**	**NAb titers**	**responders**	**non-responders**	**p**
**BS2**	51.3±14.5	54.4±14.9	NS	**BS2**	61±3.6	53.5±14.9	NS
**BS3**	61.4±15.9	52.5±14.9	0.042	**BS3**	57.2±10.7	56.7±16.8	NS
**BS4**	58.5±15.4	50±18.1	NS	**BS4**	61.1±14.3	49.5 ± 13.2	0.04
**BS5**	58.1±17	48±16.7	NS	**BS5**	59.2±15.8	51.4±19.4	NS
**BS6**	60±13.8	46.3±14.6	0.039	**BS6**	61±12.3	49.5±13.2	0.04
**BS7**	60.1±14.3	-	-	**BS7**	61.1±14.6	48.5±4.9	NS

**HDV (months)**							
**Anti-RBD Ab titers**	**responders**	**non-responders**	**p**	**NAb titers**	**responders**	**non-responders**	**p**
**BS2**	3.5(38)	26.5(47)	NS	**BS2**	3.5(-)	21(46)	NS
**BS3**	17.5(42)	18.5(50)	NS	**BS3**	3(7)	22.5(44)	0.038
**BS4**	15.5(38)	21(61)	NS	**BS4**	32(-)	15.5(44)	NS
**BS5**	24(42)	41(54)	NS	**BS5**	14.5(37)	50.5(77)	0.043
**BS6**	24(42)	23(74)	NS	**BS6**	24(39)	25(70)	NS
**BS7**	33.5(58)	-	-	**BS7**	28(39)	41(-)	NS

Abbreviations: Anti-RBD Abs, anti-receptor binding domain antibodies; BS, blood sampling; eGFR, estimated glomerular filtration rate; HDV, hemodialysis vintage; Nabs, neutralizing antibodies; and NS, non-significant.

Supplementary Table S6. Anti-SARS-CoV-2 antibody development with regards to MPA treatment

	**anti-RBD Abs**			**Nabs**		
	**p**	**OR (MPA+/MPA-)**	**CI**	**p**	**OR (MPA+/MPA-)**	**CI**
**BS2**	NS	-	-	0.001	0.057	0.015-0.219
**BS3**	NS	-	-	0.003	0.048	0.007-0.346
**BS4**	NS	-	-	NS	-	-
**BS5**	NS	-	-	NS	-	-
**BS6**	0.005	0.022	0.001-0.313	NS	-	-
**BS7**	NS	-	-	NS	-	-

Abbreviations: Anti-RBD Abs, anti-receptor binding domain antibodies; BS, blood sampling; CI, confidence interval; MPA, mycophenolic acid; Nabs, neutralizing antibodies; and OR, odds ratio.

Supplementary Table S7. Cell subpopulation concentrations (cells/μL) in BS1 and BS3 [NAb(-) patients in BS3]

	BS1	BS3	p (BS1 vs. BS3)
**WBC**	7850(2325)	7650(1750)	NS
**Lymphocytes**	1621.5(1198)	1861(851)	NS
**B-cells**	68(107)	66(101)	NS
**Naïve B-cells**	48.5(83)	46(72)	NS
**Transitional B-cells**	3(10)	1.5(2)	0.014
**Marginal B-cells**	10.9(12.2)	9.7(9.2)	NS
**Memory B-cells**	13(31)	18(24)	NS
**Plasmablasts**	0.85(3.4)	0(0.4)	0.003
**CD3+ T-cells**	1314(1102)	1528(877)	NS
**CD3+CD4+ T-cells**	832(584)	921(525)	NS
**Activated CD4+ T-cells**	5.5(6)	7.5(5)	NS
**CD3+CD8+ T-cells**	527.5(563)	560(322)	NS
**Activated CD8+ T-cells**	7(10)	19.5(28)	<0.001
**CD3+PD1+ T-cells**	79(94)	52(49)	0.029
**CD3-CD56+ (NK cells)**	21.1(65.5)	170.5(133.2)	<0.0001
**CD3+CD56+ (NKT) cells**	160.5(172)	143(140)	NS
**Activated NKT cells**	1(3)	2(4)	0.015
**Monocytes**	411(374)	511(281)	NS
**Activated monocytes**	211(217)	360.5(221)	0.025

Abbreviations: BS, blood sampling; CD, cluster of differentiation; NK, Natural Killer; NKT-cells, Natural Killer like T-cells; and NS, non-significant.

Supplementary Figures

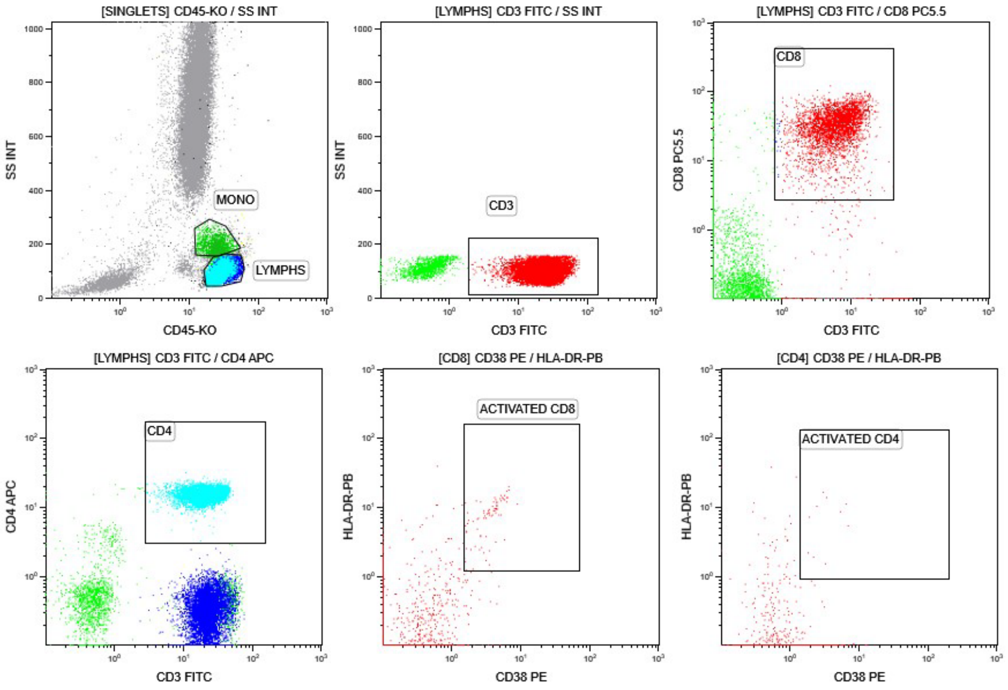

Supplementary Figure S1. Gating strategy for CD3, CD4, CD8 and Activated CD4, Activated CD8 respectively.

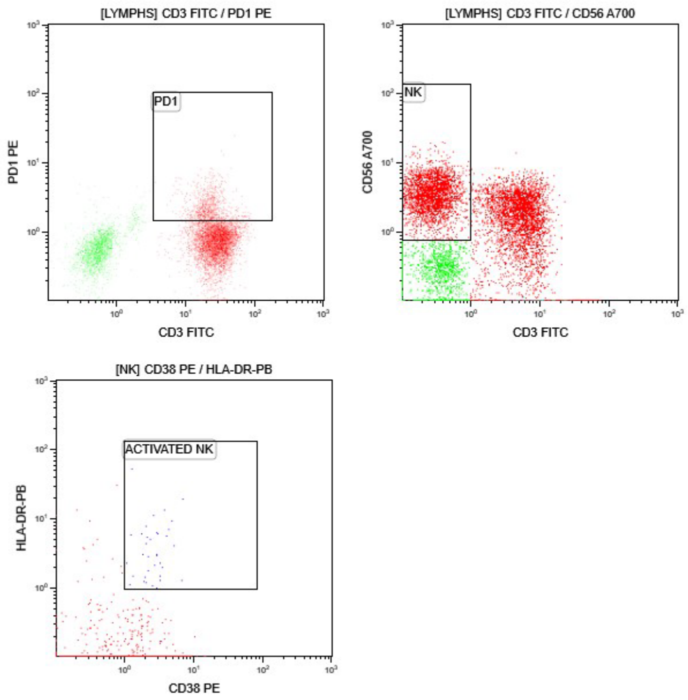

Supplementary Figure S2. Gating strategy for CD3-CD56+ (Natural Killer – NK-) cells and Activated NK cells.

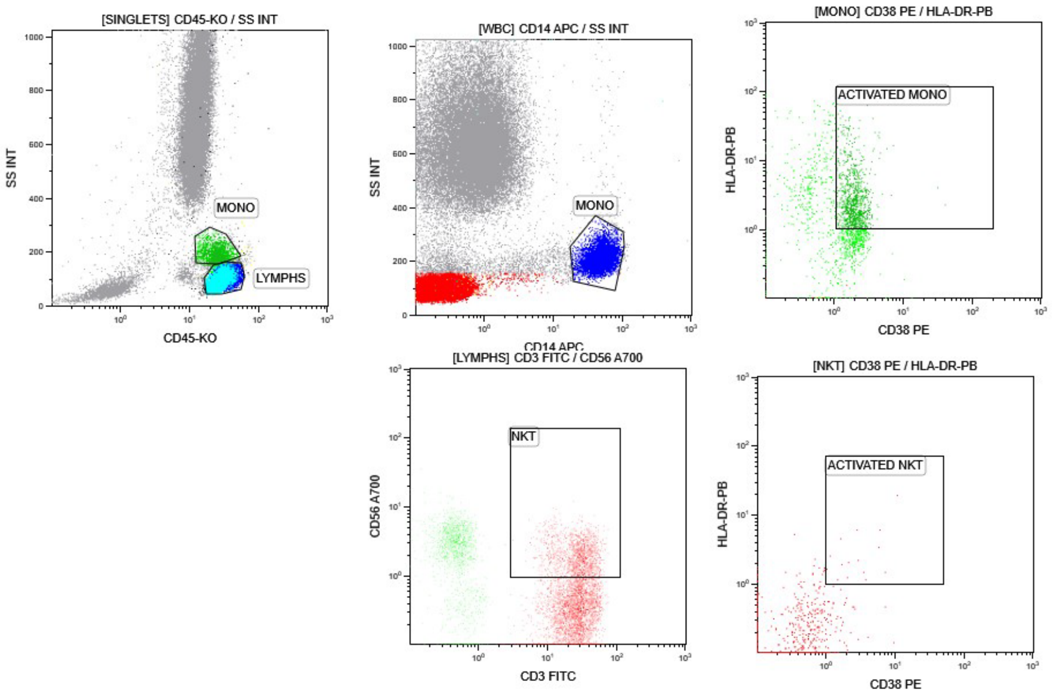

Supplementary Figure S3. Gating strategy for Monocytes and Activated Monocytes, CD3+CD56+ (NKT-cells) and Activated NKT-cells.

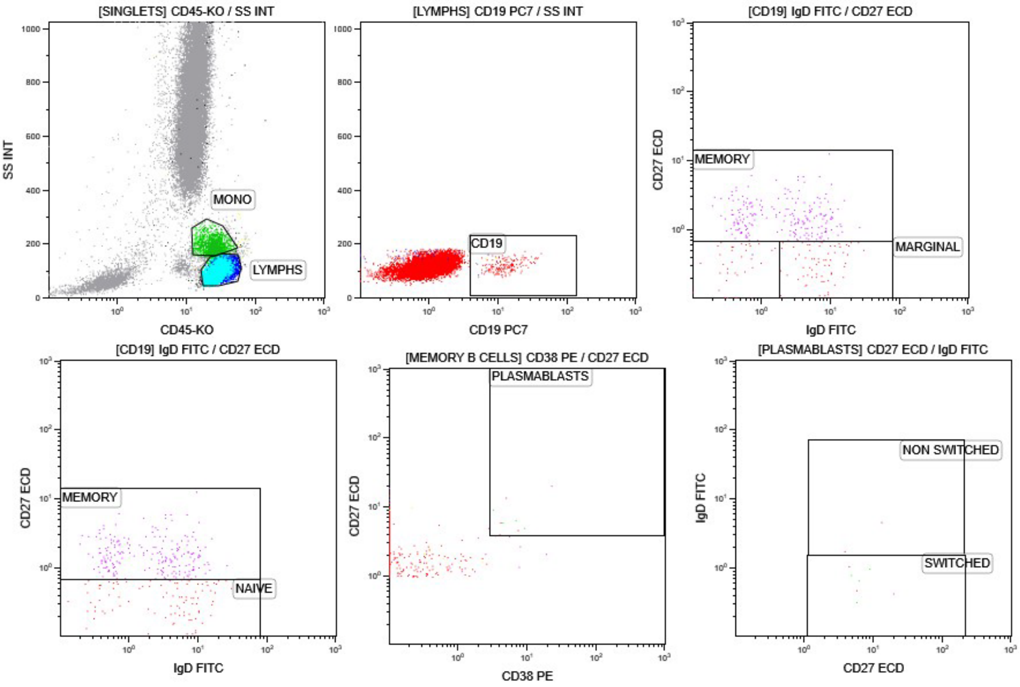

Supplementary Figure S4. Gating strategy for CD19+ cells, CD19+CD27- (naïve), CD19+CD27+ (memory), CD19+ CD27-IgD+ (marginal), CD19+CD27+CD38+(plasmablasts), CD19+CD27+IgD+ (non switched) and CD19+CD27+IgD-(switched) cells.

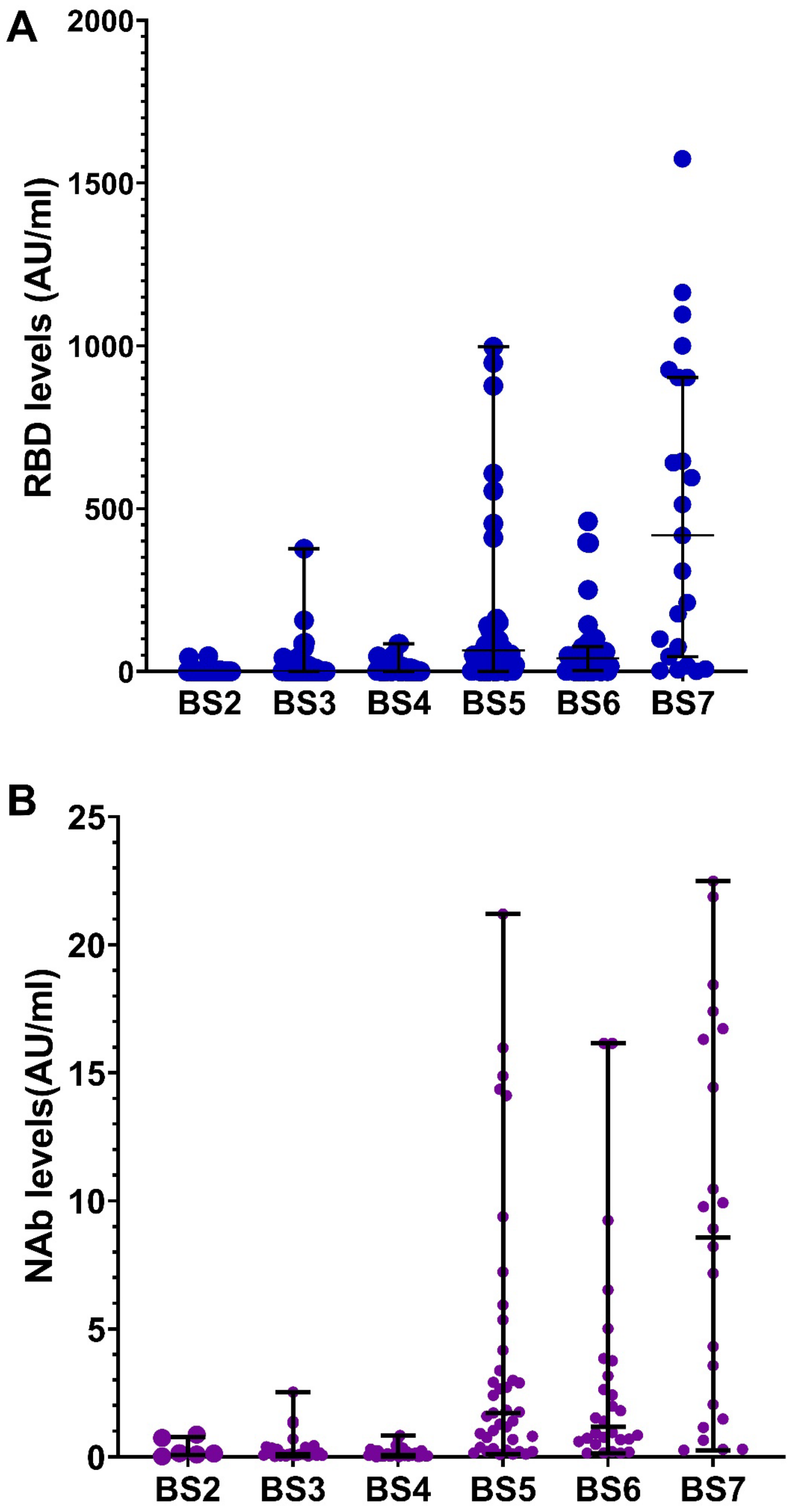
A
B
RBD levels (AU/ml)
2000
1500
1000
500
0
BS2
BS3
BS4
BS5
BS6
BS7
NAb levels(AU/ml)
25
20
15
10
5
0
BS2
BS3
BS4
BS5
BS6
BS7

Supplementary Figure S5. Serum levels of anti-RBD Abs (A) and NAbs (B) at certain time points after vaccination, during follow up, BS2, BS3, BS4, BS5, BS6, BS7. Levels at baseline, BS1, are not demonstrated because they were zero.
